# Breast Cancer and Atrial Fibrillation

**DOI:** 10.3390/jcm11051417

**Published:** 2022-03-04

**Authors:** Emanuela Mauro, Fabiana Lucà, Cecilia Tetta, Orlando Parise, Iris Parrini, Gianmarco Parise, Carmelo Massimiliano Rao, Francesco Matteucci, Linda Renata Micali, Michele Massimo Gulizia, Mark La Meir, Sandro Gelsomino

**Affiliations:** 1 Cardiothoracic Department, CARIM School for Cardiovascular Diseases, Maastricht University, 6229HX Maastricht, The Netherlands; emanuelamauroam@gmail.com (E.M.); cecilia.tetta@ior.it (C.T.); o.parise@icloud.com (O.P.); g.parise@maastrichtuniversity.nl (G.P.); melampo6@gmail.com (F.M.); l.micali@maastrichtuniversity.nl (L.R.M.); 2 Cardiology Department, Grande Ospedale Metropolitano—GMO Hospital, 89124 Reggio Calabria, Italy; fabiana.luca92@gmail.com (F.L.); massimo.rao@libero.it (C.M.R.); 3 Cardiology Department, Mauriziano Umberto I Hospital, 10128 Torino, Italy; irisparrini@libero.it; 4 Cardiology Department, Garibaldi Nesima Hospital, 95123 Catania, Italy; michele.gulizia60@gmail.com; 5 Heart Care Foundation, Via Alfonso la Marmora 36, 50121 Firenze, Italy; 6 Cardiothoracic Department, Brussels University Hospital, 1090 Brussels, Belgium; lameir@yahoo.com

**Keywords:** atrial fibrillation, breast cancer, cancer

## Abstract

This study aims to establish the incidence of atrial fibrillation (AF) in breast cancer (BC) patients, focusing on staging and anti-cancer treatment. A meta-analysis was conducted to investigate the incidence of AF in BC patients and compare this incidence to other cancers. Furthermore, we evaluated the occurrence of AF as an adverse effect of biological therapies vs. non-biological therapies vs. biological therapies + non-biological therapies in BC. Finally, we compared the incidence of AF in early BC and metastatic BC. Thirty studies were included. Twenty-two studies focused on BC, encompassing 166,271 patients. In the BC group, 2.7% of patients developed AF, while in the “all cancer” group, 5.8% of patients developed AF. In addition, there was no difference between different types of therapies (*p* = 0.61) and between early and metastatic BC (*p* = 0.57). The type of anti-cancer therapy and the staging of BC does not influence AF’s occurrence in this neoplastic disease.

## 1. Introduction

Cancer patients have a risk of atrial fibrillation (AF) 47% higher than patients without cancer [1,2,3]. Conversely, an increased risk of incidentally finding cancer in patients with known AF has also been reported so that this relationship might be bidirectional [4].

Several underlying mechanisms and risk factors are shared between these two entities [5]. Although the cause of this correlation is still not fully understood, it has been hypothesized that inflammation might play a crucial role in this association [6]. Additionally, cancer treatments might predispose to AF [7]. Patients with breast cancer (BC), the most frequent women’s tumor [8], have an increased risk of cardiovascular diseases (CVD) [9], and, among them, AF has been often reported in BC [10]. It has been hypothesized that the female sex might be a significant AF predisposing factor [4,11]. Moreover, apart from the direct effects of cancer and shared risk factors between the two diseases [6], adverse effects of cancer therapy [12] have been evoked to explain the association between AF and BC [13,14].

Nonetheless, while numerous studies have suggested a higher risk of cancer following an AF diagnosis [15] with a significant prevalence of colorectal cancer [16], there is a paucity of research in the literature focusing on AF in patients with preexisting BC [17]. In addition, despite the well-known cardiotoxic side effects of treatments frequently used in BC [18,19,20], including radiotherapy [10], little is known whether cardiotoxic chemotherapy may enhance the link between BC and AF [5,21]. Finally, the higher risk of AF has been more commonly reported in the early stage of the disease [22], whereas AF associated with metastatic breast cancer (MBC) has been rarely reported.

Therefore, this study investigates the incidence of AF following BC cancer and explores whether this association is related to BC anti-cancer treatment and whether it varies depending on BC staging.

## 2. Materials and Methods

### 2.1. Search Strategy

A literature search was performed in conformity with the principles of the Preferred Reporting Items for Systematic Review and Meta-Analyses (PRISMA) [23]. Two authors (EM and FL) decided on the search strategy approved by another author (CT). Additional references identified through original articles were reviewed manually and cross-checked for other relevant reports. Titles and abstracts of all articles published in January 2005 and August 2020 were initially screened. One investigator (EM) with great expertise in performing the literature search, performed queries, and focused on detecting articles regarding cancer and AF.

We searched PubMed, Medline, EMBASE databases. The search strategy included the following search terms: “atrial fibrillation” AND cancer, (“atrial fibrillation” AND “breast cancer”) AND (chemotherapy [MeSH Terms]), “breast cancer” AND “cardiac toxicity *,” (cancer OR “malignant neoplasm” OR neoplasm OR tumor) AND “atrial fibrillation” OR AF; (“breast cancer” OR “breast carcinoma” OR “mammary cancer”) AND “atrial fibrillation,”.

### 2.2. Selection Criteria

Article selection was based on the following inclusion criteria: (a) studies with cohorts of more than 10 patients; (b) comparative studies (BC vs. no-BC) with data on the AF incidence in a cancer population, (c) non-comparative studies on BC (BC-only).

The exclusion criteria were: (a) non-human studies, (b) case reports, (c) previous reviews and meta-analyses, (d) editorials, (e) studies consisting of less than 10 individuals, (f) studies reporting a post-operative AF, (e) studies in which data was not separated for AF and other atrial arrhythmias, (f) studies focused on BC following AF.

### 2.3. Endpoints and Definitions

The primary endpoints of this meta-analysis are: (1) To investigate the incidence of AF following BC. (2) The evaluation of the occurrence of AF during biological therapies vs. non-biological therapies (i.e., chemotherapy, radiotherapy, and hormone therapy (HT) vs. biological therapies + non-biological therapies in BC); (3) comparison between early breast cancer (EBC) and metastatic breast cancer (MBC) in terms of AF incidence. As expressed by the Union for International Cancer Control [24], we categorized EBC as (a) stage I–IIIA BC in case no specific description was reported by the study; (b) stage I–III BC, which was referred to as “early” in the study; (c) stage I–III BC for studies in which the absence of MBC was explicitly reported. We categorized MBC as BC at the IV stage [24].

### 2.4. Assessment of the Risk of Bias

The risk of bias for the included studies was independently assessed by two reviewers (EM and FL). The ROBINS-I tool (Risk of Bias in Non-randomized Studies-of Interventions) and ROB 2 tool (Risk of Bias for Randomized Trials) were used for the assessment of bias in non-randomized and randomized studies, respectively [25]. The domains assessed using the ROBINS-I tool were (1) bias due to confounding; (2) bias in the selection of participants into the study; (3) bias in classification of interventions; (4) bias due to deviations from intended interventions; (5) bias due to missing data; (6) bias in the measurement of outcomes; (7) bias in the selection of the reported result; and (8) overall bias assessment. The domains were graded as ‘Low”, “Moderate,” “Serious,” “Critical,” and “No Information” [26].

The domains assessed in the ROB 2 tool were (1) bias arising from the randomization process; (2) bias due to deviations from intended interventions; (3) bias due to missing outcome data; (4) bias in the measurement of the outcome; (5) bias in the selection of the reported result; and (6) overall bias assessment. We graded each domain as “Low,” “Some concerns,” and “High risk” [27]. Additionally, the plots for ROBINS-I and ROB 2 tools were generated using the software robvis [28].

### 2.5. Statistical Analysis

The meta-analysis was conducted using v. 3.6.1 (R Foundation for Statistical Computing, Vienna, Austria). Risk ratio (RR) and proportion with 95% confidence interval (CI) were used as index statistics for dichotomous variables. The random-effects model was employed because heterogeneity among studies was anticipated. Heterogeneity was evaluated with Higgin’s I2 test [29]. I2 values < 40% were considered having low heterogeneity, I2 values > 75% were considered having high heterogeneity. Publication bias was assessed using Egger’s test of the intercept.

A multiple (stratified) analysis was conducted. Firstly, the analysis was carried out in studies at low risk of bias, followed by a high risk of bias and all studies together, to test whether the risk of bias impacted results.

In addition, a sub-analysis was performed to explore the impact of therapies and cancer spread on the proportion of AF recurrence. *p* values < 0.05 were considered statistically significant.

## 3. Results

### 3.1. Search Results and Characteristics of the Studies

**Figure 1:**
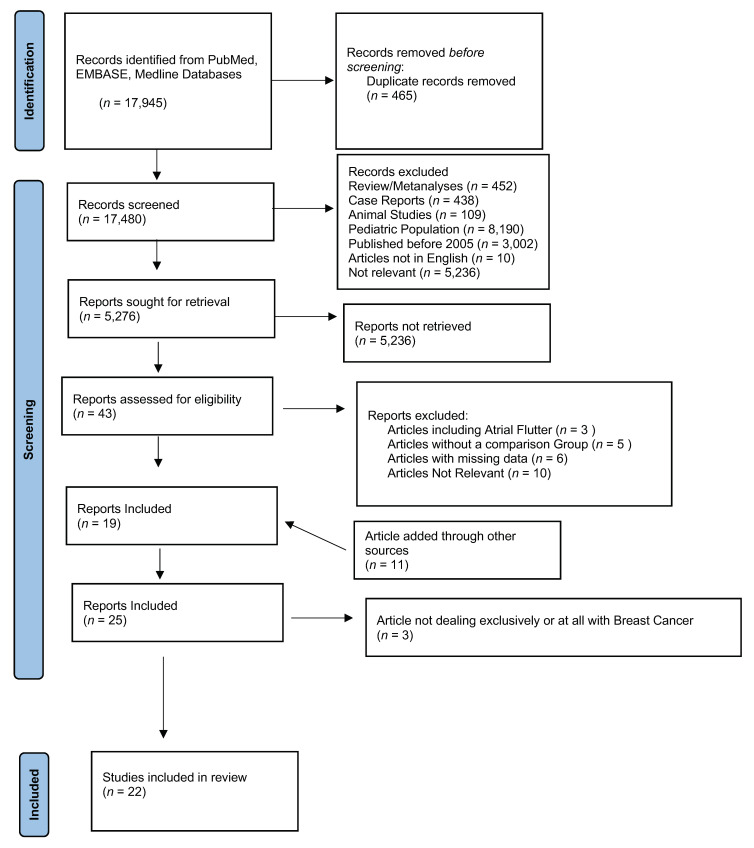
Selection of studies.

The selection process is shown in Figure 1. The Prisma checklist can be found in the Supplementary Materials (Table S1). The studies included are listed in Table 1 Since the variables needed to analyze the endpoints were not unanimously described in all papers, the variables investigated by each analysis are listed in Table 2. Twenty-two studies focused on BC, encompassing 166,271 patients [10,12,22,30,31,32,33,34,35,36,37,38,39,40,41,42] further excluding three more papers not specifically dealing with BC [22,38,42]. The mean age of BC patients was 56.88 years [95% CI: 52.93, 60.83]. Among the studies that did report information on the stage of BC, we found that 4232 patients had MBC, and 84,042 patients had EBC. Table 3 reports the characteristics of BC, while detailed information on the therapeutic strategies is presented in Table 4. The risk of bias evaluation is shown in Supplementary Materials Figures S1 and S2.

### 3.2. AF following Breast Cancer

**Figure 2:**
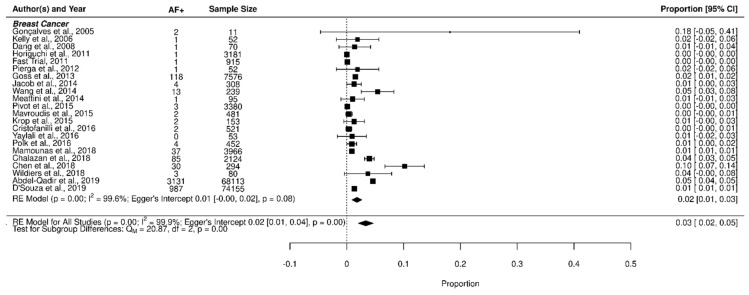
Forest plot of AF incidence in breast cancer and in other types of cancer.

In the BC group, 2.7% of patients developed AF Figure 2. In the stratified analysis, the incidence of AF in BC was comparable in patients with low and high risk of bias compared to the overall population (3% [95% CI 2–3) vs. 3% [95% CI 1–3], in low and high risk of bias, respectively) showing that the risk of bias did not impact results (Supplementary Materials Figure S3).

### 3.3. AF and Anti-Cancer Therapy

**Figure 3:**
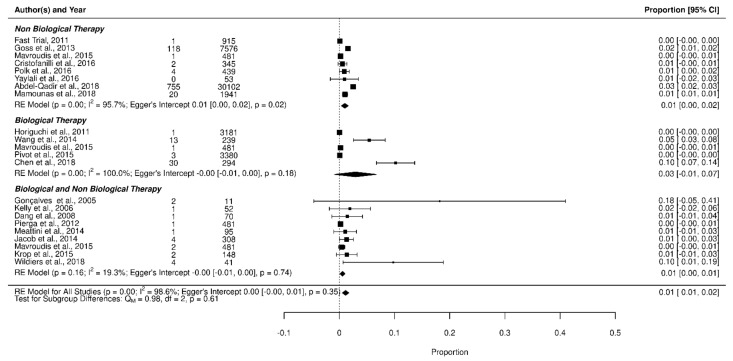
Forest plot of AF incidence in non-biological therapy vs. biological therapy vs. biological + non-biological therapy.

We found no statistically significant difference in the occurrence of AF in BC patients undergoing biological therapy, non-biological therapy, or their association; as shown in Figure 3, non-biological therapies had a cumulative AF incidence of 1% [95% CI: 0%, 2%], with 2.1% of patients developing AF while biological treatment showed a cumulative AF incidence of 3% [95% CI: −1%, 7%], with 0.6% of patients developing AF. For biological therapy associated with non-biological treatment, we reported a cumulative AF incidence of 1% [95% CI: 1%, 2%], with 1.1% of patients developing AF. The test for sub-group differences was not significant (*p* = 0.61).

### 3.4. AF in Early vs. Metastatic Breast Cancer

**Figure 4:**
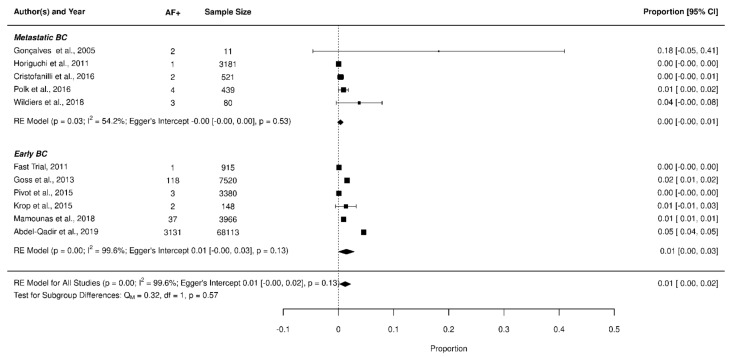
Forest plot of AF incidence Early Breast Cancer (BC) vs. Metastatic BC.

Only 0.3% of patients with MBC developed AF, leading to a cumulative incidence near to 0% [95% CI: 0–1%]. In EBC patients, 4.0% developed AF, leading to a cumulative incidence of 1% [95% CI: 0–3%]. As shown in Figure 4, there was no statistically significant difference between EBC and MBC (*p* = 0.57).

Funnel plots are reported in the Supplementary Materials (Figures S4–S6).

## 4. Discussion

In the current meta-analysis, we evaluated the incidence of AF in breast cancer (BC) patients. As far as we know this is the first meta-analysis that addresses this association related to anti-cancer therapies and cancer stages.

We estimated that the overall incidence of AF in patients with BC is 2.7%. It has been postulated that hormones play a role in the population affected by BC, in which estrogens involvement in the development/protection of AF is still controversial. Some evidence supports the antiarrhythmic role of estrogens, which could directly act on the electrical conduction of cardiomyocytes [51]. The controversy arises because exogenous and endogenous hormones exert opposite effects. On the one hand, hormone replacement therapy (HRT) in estrogen monotherapy seems to be a risk factor for AF [52]. On the other hand, some studies draw attention to the role of endogenous estrogens, which seem to be protective for the heart through their effect on blood pressure, body–mass index, and LDL [53]. In this regard, it is essential to acknowledge that the presence of estrogen receptors on the heart, which protect from cytotoxic, ischemic, and hypertrophic mechanisms, prevents left ventricular hypertrophy, commonly related to AF [53]. At the same time, low levels of endogenous hormones have been associated with AF risk factors such as obesity, dyslipidemia, and hypertension [53].

Yuan and coworkers [1] found that the risk of AF is increased in cancer patients only in the first 90 days from the cancer diagnosis. They attribute this to the hyperactivation of the sympathetic nervous system because of the diagnosis-derived mental stress. In addition, at the time of diagnosis, cancer might manifest with acute phenomena such as infections, bleeding, thrombosis, and anemia, all complications that can per se induce AF [51]. Many studies found that the risk of developing AF in cancer patients beyond the 90 days of diagnosis is equal to controls [16,54]. Remarkably, in our analysis, most studies did not include patients with newly diagnosed cancer. However, it needs to be mentioned that our results could be influenced by the high heterogeneity found, as AF might be defined and detected differently in each study.

Furthermore, in our meta-analysis, we found no difference in the incidence of AF between the different therapeutic strategies used in BC. This is not surprising since literature already reports that chemotherapeutic agents, radiotherapy, and trastuzumab have been equally associated with cardiotoxicity [55,56,57]. Chemotherapy and radiotherapy have cardiotoxic effects that lead to AF, either due to LV dysfunction or the direct action on the myocardium [58]. Ultimately, changes in the myocardium can lead to electrophysiological remodeling. Chemotherapeutic drugs cause abnormalities in potassium, sodium, and calcium channels, resulting in reducing the action potential and refractory period, facilitating the sustain of AF [57]. Additionally, they activate pro-inflammatory pathways that exacerbate the inflammatory status and predispose to AF; this is particularly true for anthracyclines, while cyclophosphamide-derived AF is due to myocardial fibrosis and hypertrophy because of modifications in the cytokine pathways. Moreover, both anthracyclines and trastuzumab lead to the accumulation of reactive oxygen species (ROS, producing cell damage, apoptosis, and cardiotoxicity, culminating in AF [57].

Finally, our sub-analysis suggest that BC staging is not a predictor of therapy-induced AF. The reason behind this balanced result could be patients’ age [59]. Younger patients seem to be frequently diagnosed with a higher stage and more aggressive BC, and so more predisposed to metastasize, but their age could decrease the risk of AF, as presumably, they have fewer comorbidities at baseline. On the contrary, older patients are mostly diagnosed with EBC, which has a better prognosis [60], and so despite their baseline conditions age-related, the AF risk could be comparable to those of younger patients with MBC.

### Clinical Implications

The cornerstones of AF management are stroke prevention, heart rate control, and rhythm control [2].

Regarding anticoagulation for the prevention of stroke, two main things must be considered when treating patients with BC and AF: (I) BC itself and cancer therapies cause a prothrombotic state [52,53]. (II) BC and Chemotherapy may increase the risk of hemorrhage, with an unpredictable anticoagulant response [1].

The European Society of Cardiology Position Paper on Cancer treatments recommends the use of anticoagulants in cancer as in non-oncological patients [54].

Despite the current evidence, anticoagulants are underused associated with large and inappropriate use of antiplatelet agents for stroke prevention as described in current registries in AF [54,55].

Therefore, although embolic-hemorrhagic risk must be evaluated in any single patient with BC and AF, anticoagulants should be prescribed as in non-oncological patients.

The use of direct oral anticoagulants (DOACs) should be preferred over vitamin K antagonists (VKAs) since the control of the international normalized ratio (INR) may be challenging [56], especially during chemotherapy. Nonetheless, Pardo Sanz et al. [57] showed that BC patients treated with DOACs experienced similar rates of stroke and bleeding as those with VKAs.

The general conditions and the quality of life of patients with BC may be strongly impacted by AF. A high heart rate, as well as the loss of the atrial contribution to left ventricular filling, reduce the left ventricular output leading to hemodynamic impairment [57]. The management should be patient-specific and can be a pharmacological or an ablative treatment [58]. Rhythm control may be required in seriously symptomatic patients, particularly when a rapid impairment of ventricular function occurs, requiring urgent cardioversion. Electrical cardioversion is preferred, while anti-arrhythmic drugs may be used only in patients with no contraindications for these drugs, and if any interference with chemotherapy drugs can be excluded. Among antiarrhythmics, dronedarone has shown also anticancer effects through antagonizing the thyroid hormone receptor alpha 1 (THRα1) that seems to play a role in breast cancer progression [59].

In very selected cancer patients with a good oncologic prognosis, a catheter ablation (CA) with pulmonary vein isolation (PVI) may be considered for rhythm control. PVI in BC patients showed rates of symptom improvements, recurrent arrhythmia, and the need for repeat ablations compared to the non-cancer population, despite a higher rate of complications [60]. Nonetheless, often BC patients have more dilated LA, therefore, presumably, more remodeled atria. Therefore, PVI alone is less effective, and more extensive lesions are necessary [60].

Finally, a rate control strategy may be obtained using beta-blockers, since the multiple interferences of verapamil, diltiazem, and digitalis with chemotherapy.

This paper has some limitations that need to be addressed. Firstly, in most reports, the number of patients who underwent radiotherapy and/or endocrine therapy was unknown. Secondly, most papers did not have two groups that could be compared.

## 5. Conclusions

Patients with BC have a >2% risk of developing AF. This risk is not influenced either by the type of anti-cancer therapy or by the BC stage. The treatment of this arrhythmia should be considered to improve the quality of life of these patients and to prevent the risk of stroke. Further specific studies are necessary to lead to specific prediction scales and precise recommendations in these patients.

## Figures and Tables

**Table 1:** Study characteristics.

Author	Year	Design	Pts	Age	Stage	Therapy	FU (mo)
Gonçalves et al. [33]	2005	PS	11	50.6 ± 30.4	IV	BT+ CT	-
Kelly et al. [35]	2006	PhII Trial	52	47 ± 33.3	IIB–IV	BT+ CT	39.0 ± NS
Dang et al. [32]	2008	PhII NRT	70	49.3 ± 33.3	-	BT + CT	29.3 ± NS
Fast Trial [43]	2011	RT	915	62.9 ± 7.2	I–III *	RTx	37.3 [NS]
Horiguchi et al. [44]	2011	RS	3181	58.1 ± 9.3	IV	BT	-
Pierga et al. [39]	2012	PhII NRT	52	48.4 ± 32.4	I–II (T4d) ^†^	BT + CT	60 ^‡^
Goss et al. [45]	2013	PhIII RCT	7576	64.1 ± NS	I–III *	HT	49.0 [NS]
Jacob et al. [34]	2014	PS	308	53.3 ± 42.9	I–III *	BT + RTx	63.1 ± 83.7
Meattini et al. [37]	2014	-	95	53.3 ± 34.8	I–III	BT + RT	5.3 ± 6.7
Wang et al. [41]	2014	-	239	71.6 ± NS	I–III	BT	50.0 [NS]
Krop et al. [36]	2015	PS	153	-	I–III *	BT + CT	24.6 [NS]
Mavroudis et al. [46]	2015	RT	481	52.3 ± 35.5	I–III *	CT/BT	47.0 [NS] 51.0 [NS]
Pivot et al. [47]	2015	PhIII RT	3380	-	I–III *	BT	53.5 ± 9.9
Cristofanilli et al. [48]	2016	PhIII RCT	521	57.2 ± 41.2	IV	CT + HT	8.9 ± 0.4
Polk et al. [40]	2016	PS	452	59.7 ± 44.4	IV	CT	-
Yaylali et al. [12]	2016	RS	53	48 ± 8.0	-	CT	-
Chalazan et al. [30]	2018	RS	2124	58.9 ± 12.8	-	CT	-
Chen et al. [31]	2018	RS	294	71.5 ± NS	I–III	BT	-
Mamounas et al. [49]	2018	PhIII RCT	3966	-	I–IIIA	HT	81.0 [73.0–89.0]
Wildiers et al. [50]	2018	PhII RT	80	78.1 ± 10.0	IV	BT + CT	20.7 [12.5–30.4]
Abdel-Qadir et al. [21]	2019	RS	68,113	60 ± 13.0	I–III	CT	67.0 ± 33.0
D’Souza et al. [10]	2019	RS	74,155	62 ± 13.3	-	-	-

Values are expressed as number or mean ± SD or median [IQR]. Abbreviations: BT = Biological Therapy, CT = Chemotherapy, FU = Follow-Up, HT = Hormone Therapy, NRT = Non-Randomized Trial, Ph = Phase, PS = Prospective Study, Pts = Patients, RCT = Randomized Controlled Trial, RS = Retrospective Study, RT = Randomized Trial, RTx = Radiotherapy. * I–III: stage reported as early-stage breast cancer, ^†^ T4d: inflammatory breast cancer, ^‡^ maximum follow-up.

**Table 2:** Variables reported in each study.

Author	Year	Variables Reported
Gonçalves et al. [33]	2005	Incidence of AF in breast cancer Incidence of AF in MBC Incidence of AF in biological therapy + chemotherapy (combined biological therapy)
Kelly et al. [35]	2006	Incidence of AF in breast cancer Incidence of AF in biological therapy + chemotherapy (combined biological therapy)
Dang et al. [32]	2008	Incidence of AF in breast cancer Incidence of AF in biological therapy + chemotherapy (combined biological therapy)
Fast Trial [43]	2011	Incidence of AF in breast cancer Incidence of AF in EBC Incidence of AF in non-biological therapies
Horiguchi et al. [44]	2011	Incidence of AF in breast cancer Incidence of AF in MBC Incidence of AF in biological therapy
Pierga et al. [39]	2012	Incidence of AF in breast cancer Incidence of AF in biological therapy + chemotherapy (combined biological therapy)
Goss et al. [45]	2013	Incidence of AF in breast cancer Incidence of AF in EBC Incidence of AF in non-biological therapies
Jacob et al. [34]	2014	Incidence of AF in breast cancer Incidence of AF in biological therapy + radiotherapy (combined biological therapy)
Meattini et al. [37]	2014	Incidence of AF in breast cancer Incidence of AF in biological therapy + radiotherapy (combined biological therapy)
Wang et al. [41]	2014	Incidence of AF in breast cancer Incidence of AF in biological therapy *
Krop et al. [36]	2015	Incidence of AF in breast cancer Incidence of AF in EBC Incidence of AF in biological therapy + chemotherapy (combined biological therapy)
Mavroudis et al. [46]	2015	Incidence of AF in breast cancer Incidence of AF in biological therapy Incidence of AF in biological therapy + chemotherapy (combined biological therapy) Incidence of AF in non-biological therapies
Pivot et al. [47]	2015	Incidence of AF in breast cancer Incidence of AF in EBC Incidence of AF in biological therapy
Cristofanilli et al. [48]	2016	Incidence of AF in breast cancer Incidence of AF in MBC Incidence of AF in non-biological therapies
Polk et al. [40]	2016	Incidence of AF in breast cancer Incidence of AF in MBC Incidence of AF in non-biological therapies
Yaylali et al. [12]	2016	Incidence of AF in no-cancer vs. cancer patients Incidence of AF in breast cancer Incidence of AF in non-biological therapies
Chalazan et al. [30]	2018	Incidence of AF in breast cancer Incidence of AF in non-biological therapies
Chen et al. [31]	2018	Incidence of AF in breast cancer Incidence of AF in biological therapy
Mamounas et al. [49]	2018	Incidence of AF in breast cancer Incidence of AF in EBC Incidence of AF in non-biological therapies
Wildiers et al. [50]	2018	Incidence of AF in breast cancer Incidence of AF in MBC Incidence of AF in biological therapy + chemotherapy (combined biological therapy)
Abdel-Qadir et al. [21]	2019	Incidence of AF in no-cancer vs. cancer patients Incidence of AF in breast cancer Incidence of AF in EBC Incidence of AF in non-biological therapies
D’Souza et al. [10]	2019	Incidence of AF in no-cancer vs. cancer patients Incidence of AF in breast cancer

The studies are ordered chronologically. Abbreviations: AF = Atrial Fibrillation, EBC = Early Breast Cancer, MBC = Metastatic Breast Cancer. * Early discontinuation of trastuzumab.

**Table 3:** Breast cancer characteristics.

Author	Year	HER2 Status	N Status	HR Status
Gonçalves et al. [33]	2005	HER2+: 11 (100) HER2−: 0 (0)	N+: 7 (63.6)	HR+ *: 6 (54.5)
Kelly et al. [35]	2006	HER+: 52 (100) HER2−: 0 (0)	-	ER+: 24 (46.0)
Dang et al. [32]	2008	HER2+: 32 (46.0) HER−: 38 (54.0)	-	ER+: 32 (46.0) PR+: 23 (33.0)
Fast Trial [43]	2011	-	-	-
Horiguchi et al. [44]	2011	HER2+: 3181 (100) HER2−: 0 (0)	-	-
Pierga et al. [39]	2012	HER+: 52 (100) HER2−: 0 (0)	N−: 14 (27.0) N+: 37 (71.2) Unknown: 1 (2.0)	-
Goss et al. [45]	2013	-	N−: 5371 (70.9) N+ †: 2206 (29.1)	ER+: 7525 (99.3) PR+: 6090 (80.4)
Jacob et al. [34]	2014	HER+: 308 (100) HER2-: 0 (0)	N−: 175 (58.8) N+:133 (43.2) Unknown:1 (0.3)	HR+ *: 165 (53.6)
Meattini et al. [37]	2014	HER2+: 95 (100)	N−: 35 (36.9) N+: 60 (63.2)	ER+: 60 (63.1) PR+: 47 (48.5)
Wang et al. [41]	2014	HER2+: 239 (100) HER2−: 0 (0)	N−: 112 (47.0) N+: 111 (46.4)	-
Krop et al. [36]	2015	HER2+: 153 (100) HER2−: 0 (0)	-	HR+ *: 95 (62.1)
Mavroudis et al. [46]	2015	HER+: 481 (100) HER2−: 0 (0)	N−: 101 (21.0) N+: 380 (79.0)	HR+ *: 321 (66.7) Unknown: 1 (0.2)
Pivot et al. [47]	2015	HER2+: 3380 (100) HER2−: 0 (0)	-	-
Cristofanilli et al. [48]	2016	HER+: 0 (0) HER2−: 521 (100)	-	-
Polk et al. [40]	2016	-	-	-
Yaylali et al. [12]	2016	-	-	-
Chalazan et al. [30]	2018	-	-	-
Chen et al. [31]	2018	-	-	-
Mamounas et al. [49]	2018	HER2+: 565 (14.2) HER2−: 3093 (78.0) Unknown: 308 (7.8)	N+: 1687 (42.5) N−: 2279 (57.5)	-
Wildiers et al. [50]	2018	HER2+: 80 (100) HER2−: 0 (0)	-	HR+ *: 55 (68.8)
Abdel-Qadir et al. [21]	2019	-	-	-
D’Souza et al. [10]	2019	-	-	-

Values are expressed as n (%). Abbreviations: ER = Estrogen Receptor, HER2 = Human Epidermal Growth Factor Receptor 2, HR = Hormonal Receptor, N = Nodal, PR = Progesterone Receptor. * Estrogen/progesterone receptor or both, † Positive lymph nodes/missing data.

**Table 4:** Therapeutic strategies.

Author	Year	Biological Therapy	Chemotherapy	Radiotherapy	Endocrine Therapy
Gonçalves et al. [33]	2005	Trastuzumab: 11 (100)	Alkylating agents: 11 (100)	No	No
Kelly et al. [35]	2006	Trastuzumab: 52 (100)	AC regimen + antimicrotubule agents (paclitaxel): 52 (100)	No	No
Dang et al. [32]	2008	Trastuzumab: 70 (100)	AC regimen + antimicrotubule agents (paclitaxel): 70 (100)	No	No
Fast Trial [43]	2011	No	No	Yes	No
Horiguchi et al. [44]	2011	Trastuzumab: 3181 (100)	No	No	No
Pierga et al. [39]	2012	Bevacizumab: 52 (100) *,† Trastuzumab: 52 (100) *,†	Antimetabolites (fluorouracil): 52 (100) * Anthracyclines (epirubicin): 52 (100) * Alkylating agents (cyclophosphamide): 52 (100) * Antimicrotubule agents (docetaxel): 52 (100) *	Yes†	Yes ‡
Goss et al. [45]	2013	Trastuzumab: 74 (1.0)	No	No	Aromatase inhibitors: 7576 (100)
Jacob et al. [34]	2014	Trastuzumab: 308 (100)	FEC regimen + antimicrotubule agents (paclitaxel): 293 (95.1)	Yes	Aromatase inhibitors: 76 LHRH agonists: 42 Tamoxifen: 30 Aromatase inhibitors + LHRH agonist: 9
Meattini et al. [37]	2014	Trastuzumab: 95 (100)	Anthracyclines (epirubicin, doxorubicin): 91 (95.8)	Yes	Aromatase inhibitors: 50 (52.6) LHRH agonist/ Tamoxifen: 11 (11.6)
Wang et al. [41]	2014	Trastuzumab: 585 (100)	AC regimen + antimicrotubule agents (paclitaxel and docetaxel): 585 (100)	No	No
Krop et al. [36]	2015	Trastuzumab emtansine: 148 (96.7) Trastuzumab: 74 (48.4)	AC regimen + antimicrotubule agents (docetaxel): 68 (44.4) FEC regimen + antimicrotubule agents (docetaxel): 84 (54.9)	Yes	62 (40.5)
Mavroudis et al. [46]	2015	Trastuzumab: 481 (100) ^†^	FEC regimen + antimicrotubule agents (docetaxel): 481 (100) †	Yes ‡	Yes ‡
Pivot et al. [47]	2015	Trastuzumab: 3380 (100)	Yes ‡	Yes ‡	Yes ‡
Cristofanilli et al. [48]	2016	No	No	No	Fulvestrant + palbociclib: 191 (78.9) Fulvestrant: 51 (21.1)
Polk et al. [40]	2016	Trastuzumab: 54 (12.0)§	Antimetabolites (capecitabine): 452 (100) Anthracyclines: 242 (54.0) ^§^	No	No
Yaylali et al. [12]	2016	No	AC regimen + antimicrotubule agents (paclitaxel): 53 (100)	No	No
Chalazan et al. [30]	2018	Monoclonal antibodies	Tyrosine kinase inhibitors Alkylating agents Antimetabolites Mitotic inhibitors Topoisomerase inhibitors Antineoplastic antibiotics	Yes	Hormonal modifiers
Chen et al. [31]	2018	Trastuzumab: 294 (100)	No	No	No
Mamounas et al. [49]	2018	No	No	No	Aromatase inhibitor (letrozole): 1983 (100)
Wildiers et al. [50]	2018	Pertuzumab + trastuzumab: 80 (100)	Alkylating agents (cyclophosphamide): 41 (51.3)	Yes ‡	Yes ‡
Abdel-Qadir et al. [21]	2019	Trastuzumab: 8365 (12.3)	Anthracyclines or other chemotherapy: 36,222 (53.2)	48,816 (71.7)	No
D’Souza et al. [10]	2019	-	-	-	-

Values are expressed as *n* (%). Abbreviations: AC = Doxorubicin plus Cyclophosphamide, FEC = Fluorouracil plus Epirubicin plus Cyclophosphamide, LHRH = Luteinizing Hormone-Releasing Hormone. * Neoadjuvant, † Adjuvant, ‡ At the investigator’s discretion in patients with hormone receptor-positive breast cancer (unknown number), § Previous or concurrent treatment.

## Supplementary Materials

The following supporting information can be downloaded at: https://www.mdpi.com/article/10.3390/jcm11051417/s1, Figure S1: The risk-of-bias plot for the Robins-I, Figure S2: The risk-of-bias plot for the RoB2, Figure S3: (A) Odds-Ratio forrest plot of Supraventricular Arrhytmias vs. OSA, (B) Odds-Ratio forrest plot of AF vs. OSA, Figure S4: Funnel plot of AF incidence in breast cancer and in other types of cancer, Figure S5: Funnel plot of AF incidence in non-biological therapy vs. biological therapy vs. biological + non-biological therapy, Figure S6: Funnel plot of AF incidence Early Breast Cancer (BC) vs. Metastatic BC, Table S1: The PRISMA statement.
