# Supplementary material for: Breast Cancer and Atrial Fibrillation
Source: J Clin Med. 2022 Mar 4;11(5):1417. doi: 10.3390/jcm11051417 (PMC8911432; doi:10.3390/jcm11051417)
Supplement: Supplementary file 1 [file jcm-11-01417-s001.zip › jcm-1560928-supplementary.pdf]

Supplementary Materials

Table S1. The PRISMA statement.

**PRISMA CHECKLIST**			
**Section and Topic**	**Item #**	**Checklist item**	
**TITLE**			
Title	1	Identify the report as a systematic review.	1
**ABSTRACT**			
Abstract	2	See the PRISMA 2020 for Abstracts checklist.	1
**INTRODUCTION**			
Rationale	3	Describe the rationale for the review in the context of existing knowledge.	1
Objectives	4	Provide an explicit statement of the objective(s) or question(s) the review addresses.	2
**METHODS**			
Eligibility criteria	5	Specify the inclusion and exclusion criteria for the review and how studies were grouped for the syntheses.	2
Information sources	6	Specify all databases, registers, websites, organisations, reference lists and other sources searched or consulted to identify studies. Specify the date when each source was last searched or consulted.	2
Search strategy	7	Present the full search strategies for all databases, registers and websites, including any filters and limits used.	2
Selection process	8	Specify the methods used to decide whether a study met the inclusion criteria of the review, including how many reviewers screened each record and each report retrieved, whether they worked independently, and if applicable,	2

		details of automation tools used in the process.	
Data collection process	9	Specify the methods used to collect data from reports, including how many reviewers collected data from each report, whether they worked independently, any processes for obtaining or confirming data from study investigators, and if applicable, details of automation tools used in the process.	2
Data items	10a	List and define all outcomes for which data were sought. Specify whether all results that were compatible with each outcome domain in each study were sought (e.g. for all measures, time points, analyses), and if not, the methods used to decide which results to collect.	2
	10b	List and define all other variables for which data were sought (e.g. participant and intervention characteristics, funding sources). Describe any assumptions made about any missing or unclear information.	2
Study risk of bias assessment	11	Specify the methods used to assess risk of bias in the included studies, including details of the tool(s) used, how many reviewers assessed each study and whether they worked independently, and if applicable, details of automation tools used in the process.	2
Effect measures	12	Specify for each outcome the effect measure(s) (e.g. risk ratio, mean difference) used in the synthesis or presentation of results.	3
Synthesis methods	13a	Describe the processes used to decide which studies were eligible for each synthesis (e.g. tabulating the study intervention characteristics and comparing against the planned groups for each synthesis (item #5)).	3
	13b	Describe any methods required to prepare the data for presentation or synthesis, such as handling of missing summary statistics, or data conversions.	3
	13c	Describe any methods used to tabulate or visually display results of individual studies and syntheses.	3
	13d	Describe any methods used to synthesize results and provide a rationale for the choice(s). If meta-analysis was performed, describe the model(s), method(s) to identify the presence and extent of statistical heterogeneity, and software package(s) used.	3
	13e	Describe any methods used to explore possible causes of heterogeneity among study results (e.g. subgroup analysis, meta-regression).	3

	13f	Describe any sensitivity analyses conducted to assess robustness of the synthesized results.	3
Reporting bias assessment	14	Describe any methods used to assess risk of bias due to missing results in a synthesis (arising from reporting biases).	2-3
Certainty assessment	15	Describe any methods used to assess certainty (or confidence) in the body of evidence for an outcome.	2-3
**RESULTS**			
Study selection	16a	Describe the results of the search and selection process, from the number of records identified in the search to the number of studies included in the review, ideally using a flow diagram.	4
	16b	Cite studies that might appear to meet the inclusion criteria, but which were excluded, and explain why they were excluded.	4
Study characteristics	17	Cite each included study and present its characteristics.	5-8
Risk of bias in studies	18	Present assessments of risk of bias for each included study.	8-9
Results of individual studies	19	For all outcomes, present, for each study: (a) summary statistics for each group (where appropriate) and (b) an effect estimate and its precision (e.g. confidence/credible interval), ideally using structured tables or plots.	7-8
Results of syntheses	20a	For each synthesis, briefly summarise the characteristics and risk of bias among contributing studies.	-
	20b	Present results of all statistical syntheses conducted. If meta-analysis was done, present for each the summary estimate and its precision (e.g. confidence/credible interval) and measures of statistical heterogeneity. If comparing groups, describe the direction of the effect.	-
	20c	Present results of all investigations of possible causes of heterogeneity among study results.	-
	20d	Present results of all sensitivity analyses conducted to assess the robustness of the synthesized results.	-

Reporting biases	21	Present assessments of risk of bias due to missing results (arising from reporting biases) for each synthesis assessed.	8
Certainty of evidence	22	Present assessments of certainty (or confidence) in the body of evidence for each outcome assessed.	
**DISCUSSION**			
Discussion	23a	Provide a general interpretation of the results in the context of other evidence.	11
	23b	Discuss any limitations of the evidence included in the review.	12
	23c	Discuss any limitations of the review processes used.	12
	23d	Discuss implications of the results for practice, policy, and future research.	12
**OTHER INFORMATION**			
Registration and protocol	24a	Provide registration information for the review, including register name and registration number, or state that the review was not registered.	13
	24b	Indicate where the review protocol can be accessed, or state that a protocol was not prepared.	13
	24c	Describe and explain any amendments to information provided at registration or in the protocol.	13
Support	25	Describe sources of financial or non-financial support for the review, and the role of the funders or sponsors in the review.	13
Competing interests	26	Declare any competing interests of review authors.	13
Availability of data, code and other materials	27	Report which of the following are publicly available and where they can be found: template data collection forms; data extracted from included studies; data used for all analyses; analytic code; any other materials used in the review.	13

Risk of bias assessment

Figure S1 shows the risk-of-bias plot for the Robins-I. Twenty-two studies were non-randomized, and they were evaluated with the Robins-I tool (21, 30-46). We expected to find confounding in all the non-randomized studies, and in fact, none of the papers had a low score. Some of them controlled for crushing through their statistical analysis. Five papers had low detection bias because patients seemed to be included during intervention and follow-up. Bias in the classification of intervention was low for nine papers, as intervention status was properly defined, and its definition was not obtained retrospectively. Six papers received a score of "low risk" in terms of bias due to deviations from intended interventions because the intervention switches did not affect the outcome, thereby reflecting current clinical practice. Bias due to missing data was low in six papers because either there was no missing data, or the amount of missing data was too insignificant to have influenced the outcome. Six papers had a low bias in measuring outcomes because the outcome assessed was comparable for the two groups, and the fact that blinding was not used is unlikely to have influenced its measurement. Bias in selecting the reported results was considered low in all eight papers. No study showed overall low bias.

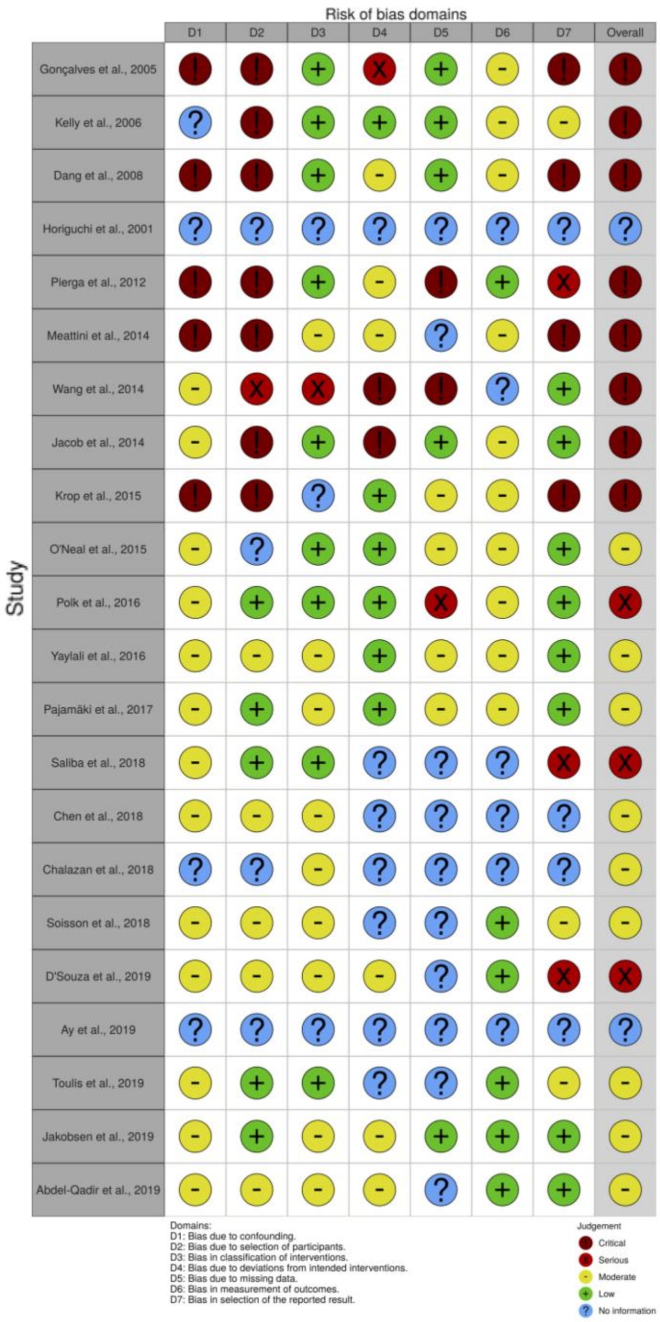

Figure S1. The risk-of-bias plot for the Robins-I

Figure S2 shows the risk-of-bias plot for the RoB2. Seven studies were randomized, and they were evaluated using the RoB 2 tool [44–50] Six studies had low bias arising from the randomization process because the allocation was concealed correctly and/or the allocation sequence was random. Bias due to deviations from intended intervention was low in three studies. Bias due to missing data was scarce in six papers, as in those papers, data were available for all patients. All studies had a low bias in the outcome measurement because the methods used for measuring outcomes were appropriate and did not differ across the intervention groups. All studies there had a low bias in selecting the reported results as the data were analyzed according to a pre-specified plan. Four studies had a low overall bias.

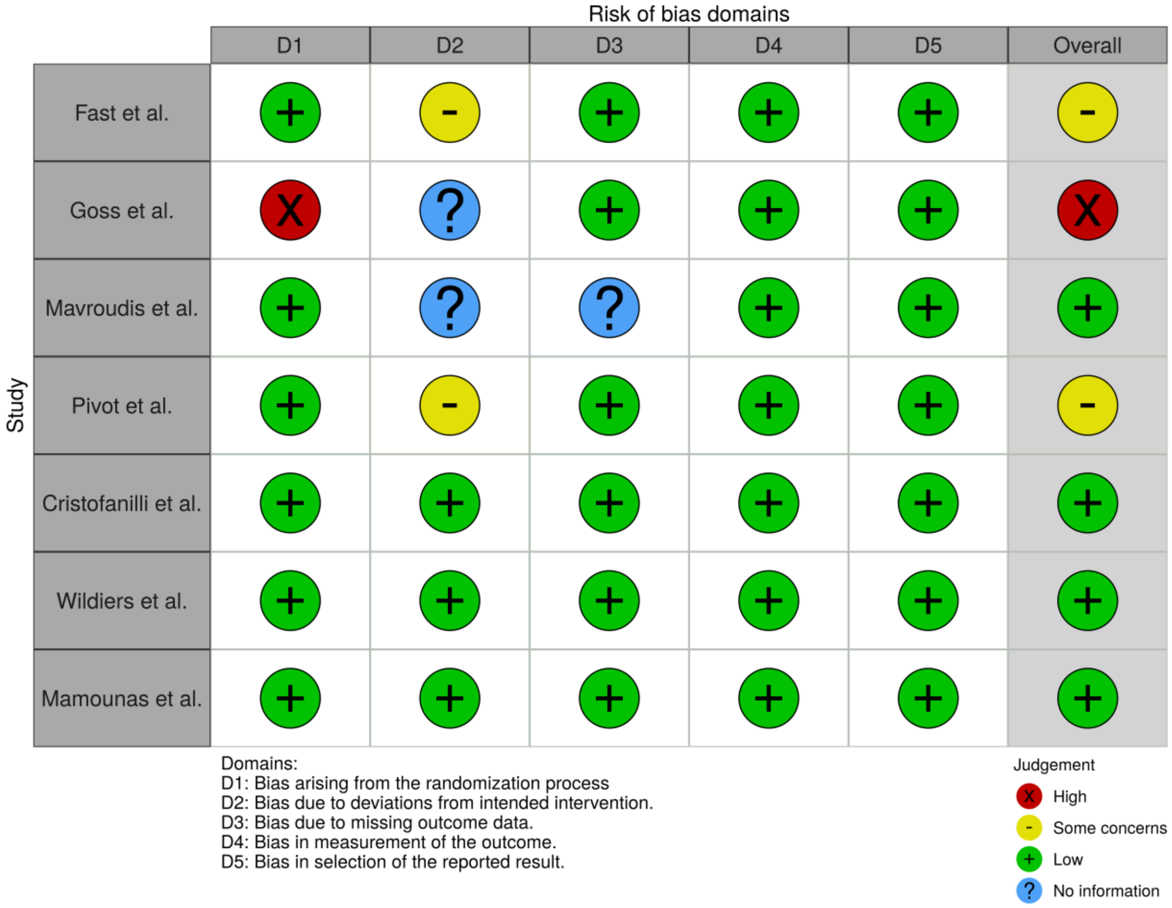

Figure S2. The risk-of-bias plot for the RoB2

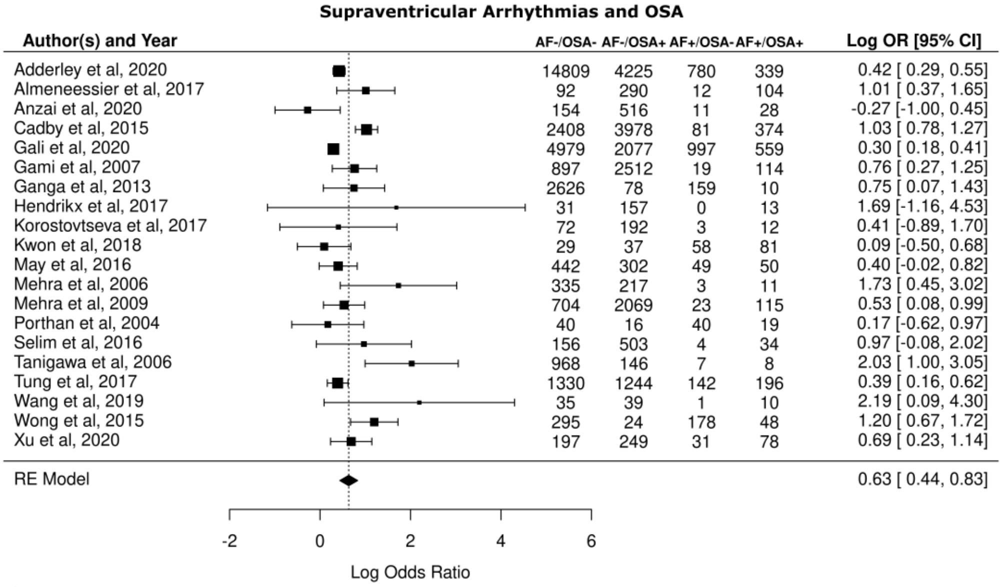

A

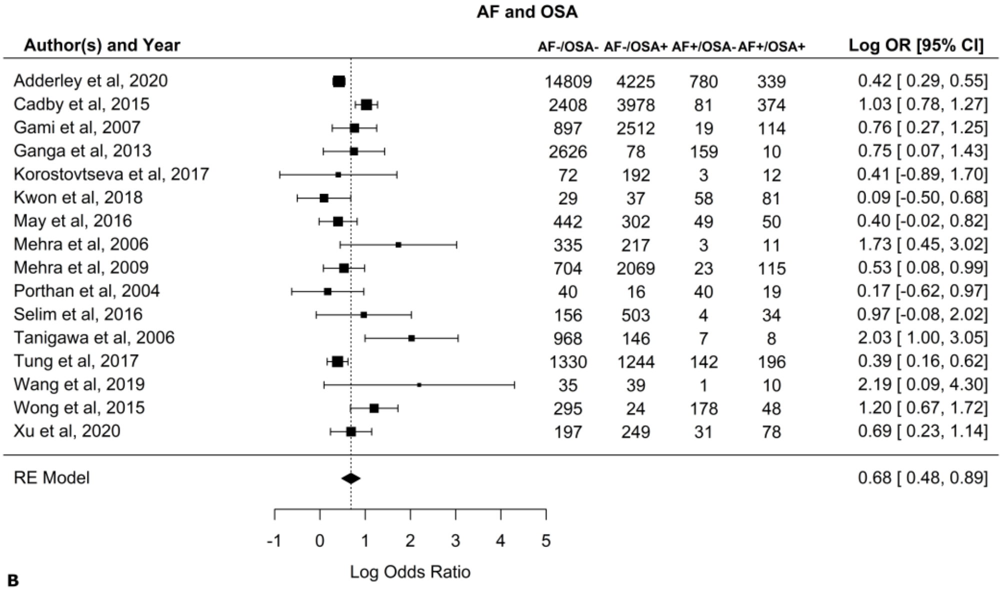

B

Figure S3. (A) Odds-Ratio forrest plot of Supraventricular Arrhytmias vs. OSA , (B) Odds-Ratio forrest plot of AF vs. OSA.

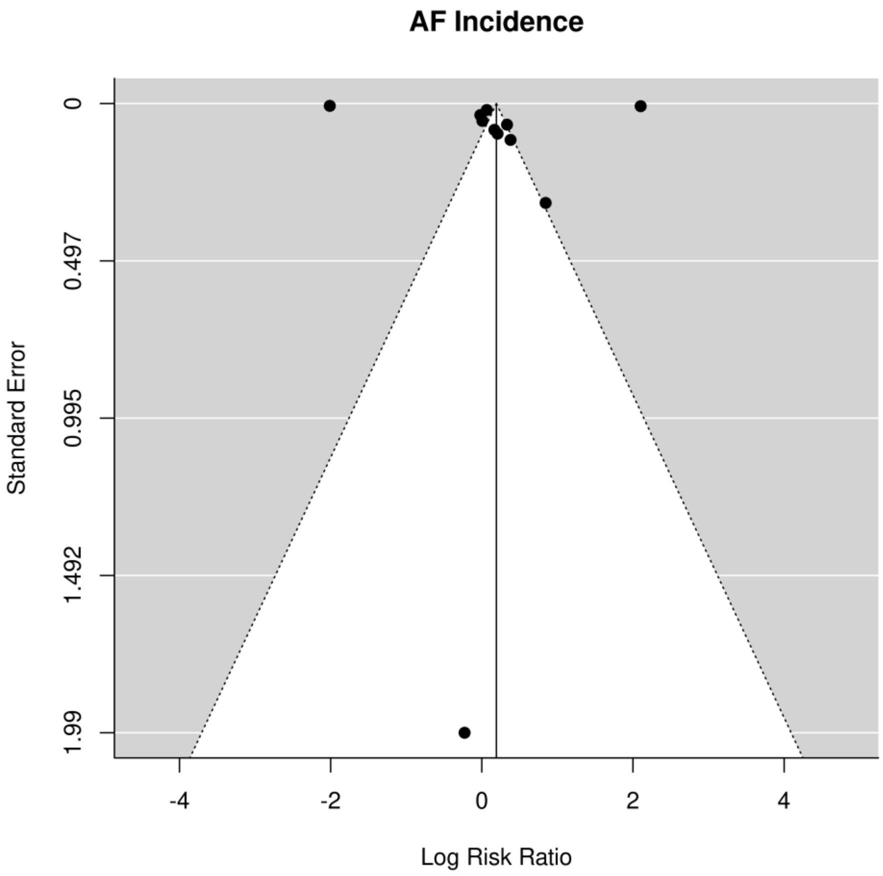

Figure S4. Funnel plot of AF incidence in breast cancer and in other types of cancer.

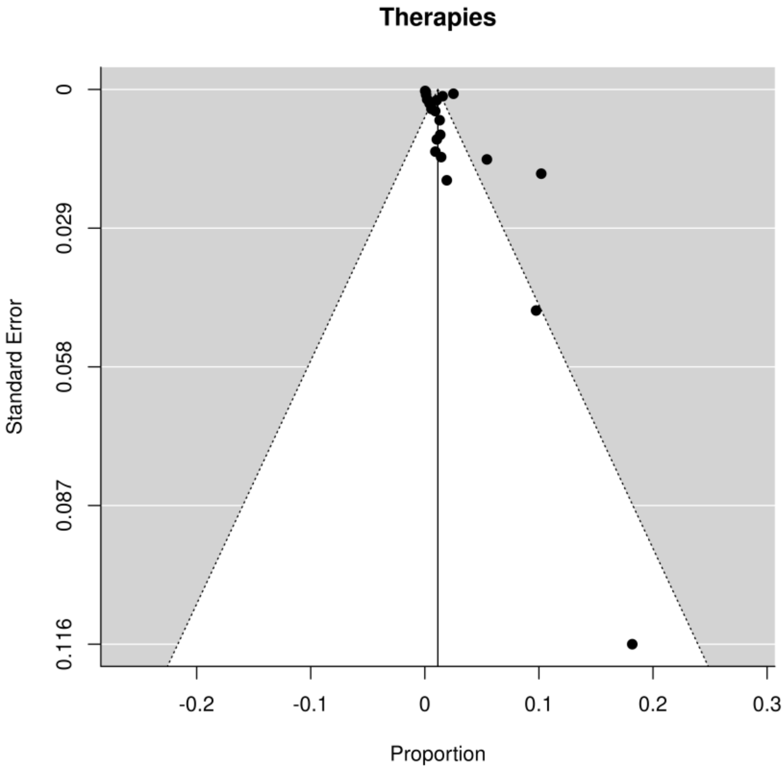

Figure S5. Funnel plot of AF incidence in non-biological therapy vs biological therapy vs biological + non-biological therapy.

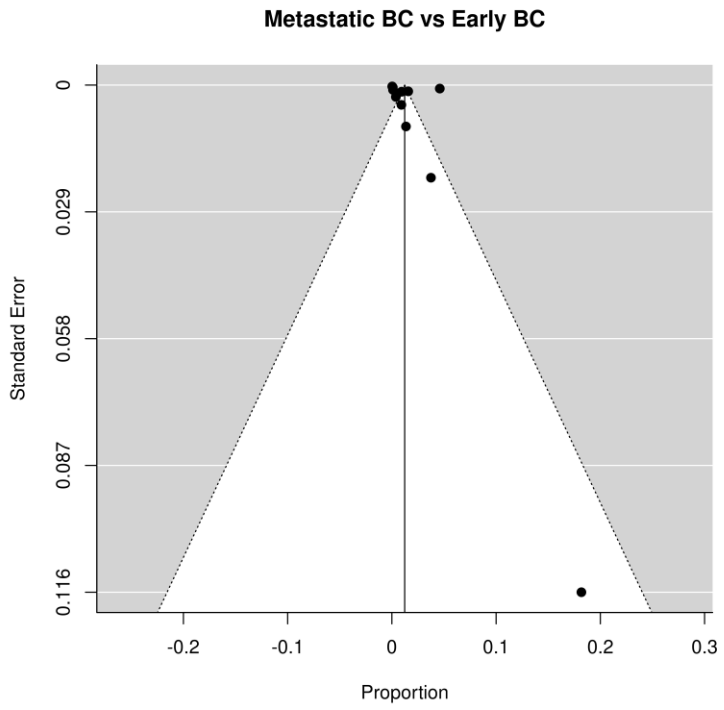

Figure S6. Funnel plot of AF incidence Early Breast Cancer (BC) vs Metastatic BC
